# Three-dimensıonal cone-beam computed tomography for diagnosıs of keratocystic odontogenic tumours; Evaluation of four cases

**DOI:** 10.4317/medoral.17629

**Published:** 2012-05-01

**Authors:** Hülya Koçak-Berberoğlu, Sırmahan Çakarer, Amila Brkić, Banu Gürkan-Koseoglu, Barış Altuğ-Aydil, Cengizhan Keskin

**Affiliations:** 1 DDS, PhD, Department of Oral and Maxillofacial Surgery, Faculty of Dentistry, Istanbul University Capa, Istanbul, Turkey; 2 DDS, PhD, Department of Oral Surgery, School of Dental Medicine, University of Sarajevo, Bosnia and Herzegovinia

## Abstract

The keratocystic odontogenic tumour (KCOT), formerly known as the odontogenic keratocyst (OKC) is a benign intraosseous lesion that derives from remnants of the dental lamina. Due to its characteristics, clinical and histopathological features and various treatment approaches, this pathology is different comparing with other odontogenic cysts. Radiographically the KCOT appears as well-defined unilocular or multilocular radiolucency with thin radiopaque borders. In most cases, conventional radiographic imaging, such as panoramic views and intraoral periapical films, are adequate to determine the location and estimate the size of an KCOT. However, the clinical use for cone-beam computed tomography (CBCT) in oral and maxillofacial surgery increases and provides additional information about the contents and borders of the large lesions. In the present cases, the diagnostic performances of CBCT versus panoramic radiograph for four KCOTs were evaluated. It was concluded that appearance of lesions in the maxillofacial region could be better documented in the correct dimensions by CBCT versus panoramic radiograph.

** Key words:**Odontogenic keratocyst, cone-beam computed tomography, three dimensional, panoramic radiograph.

## Introduction

The odontogenic keratocyst (OKC) is designated by the World Health Organization (WHO) as a keratocystic odontogenic tumour (KCOT) and is defined as “a benign uni- or multicystic, intraosseous tumour of odontogenic origin, with a characteristic lining of parakeratinized stratified squamous epithelium (1).

The potential for aggressive clinical behavior and local recurrence resulted in its recent classification as a benign odontogenic tumor (2).

A more “innocent” entity ortokeratinized odontogenic keratocyst (OKK) is not included in this novel classification, is still classified in developmental odontogenic cysts (3).

The keratocystic odontogenic tumour, which represents between 4-12% of all odontogenic cysts, is believed to arise from dental lamina remnants and extension of basal cells of overlying oral epithelium (4-6). However, by some authors, Deoxy-ribo-Nucleic Acid (DNA) mutations might be also included as the etiological factors, which is supported by malignant transformation of the KCOT cells (6). By these evidences, developmental theory of KCOT occurrence might be discarded, and changed to neoplastic theory (6).

KCOTs have a predilection for males and occur mainly in the second and third decade of life. It may occur anywhere in the jaws, but is most frequently found in the posterior region of the mandible (7,8). Multiple lesions are almost always associated with ne-void basal cell carcinoma syndrome, especially in the young patients (7).

A localized asymptomatic swelling associate with mobility of the teeth, and spontaneous drainage of the tumour into the oral cavity, are the most common symptomes of the KCOT occurrence (5,8). Radiographically, the tumour is characterized as well borded unilobular or multilobular, sometimes expansile, radiolucent lesion with smooth and usually sclerotic margins.

Traditional radiographic examinations are usually limited to two-dimensional views captured using radiographic film or digital sensors and are adequate to determine the location and estimate the size of the tumour. However, the clinical applications for cone-beam computed tomography (CBCT) imaging in the oral and maxillofacial (OMF) region are increasing. A major advantage of CBCT that has been reported is the three-dimensional geometric accuracy compared with conventional radiographs. Sagittal, coronal and axial CBCT images eliminate the superimposition of anatomical structures (9).

Despite the fact, that the most clinical applications in OMF region are reported as impacted teeth and implantology (10), it is well known that CBCT have an important role for the diagnosis of the odontogenic lesions including a keratocystic odontogenic tumour (11). The advantages of this technique are relatively high isotropic spatial resolution of osseous structures with a reduced radiation dose compared with conventional computed tomography (CT) scans, low cost and easy accessibility (10).

The purpose of this article is to present four clinical cases of KCOTs for which managements, diagnostic accuracy of the CBCT versus panoramic radiographs were compared.

## Case Section

Four patients (three male and one female), age range 24 to 56 with solitary or multiple jaw lesions, clinically and histopathologically diagnosed as the ‘‘KCOT’’, treated in the department of oral and maxillofacial surgery, Faculty of Dentistry University of Istanbul, were included in this study case presentation ( Table 1). For diagnostic managements, a fine-needle aspiration, panoramic radiographs and cone-beam computed tomography were used and compared.

**Table 1:**
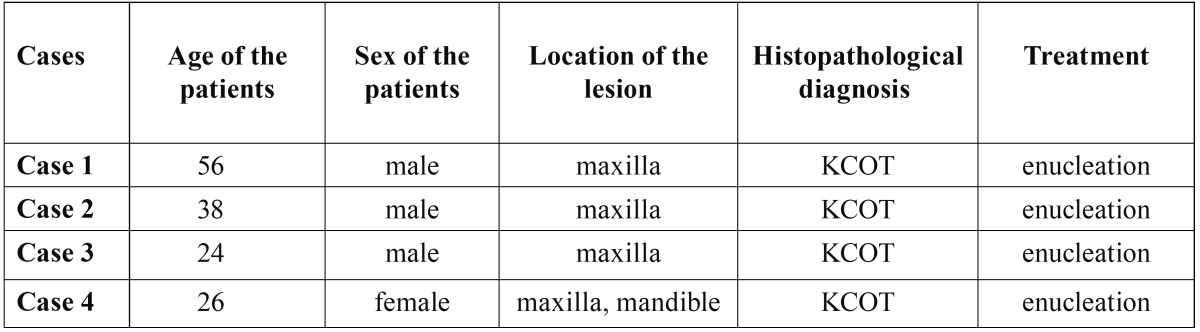
Description of the patients and the lesions.

-Case 1

**Figure 1:**
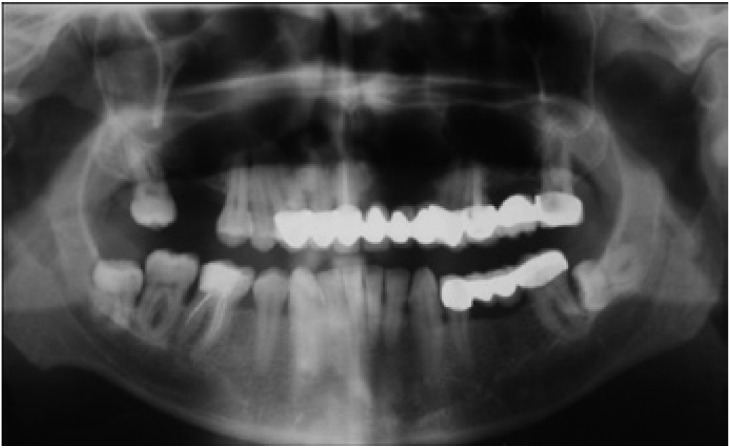
Panoramic radiograph of a KCOT occupying the right maxillary sinus. Note that the border is not readily apparent.

**Figure 2:**
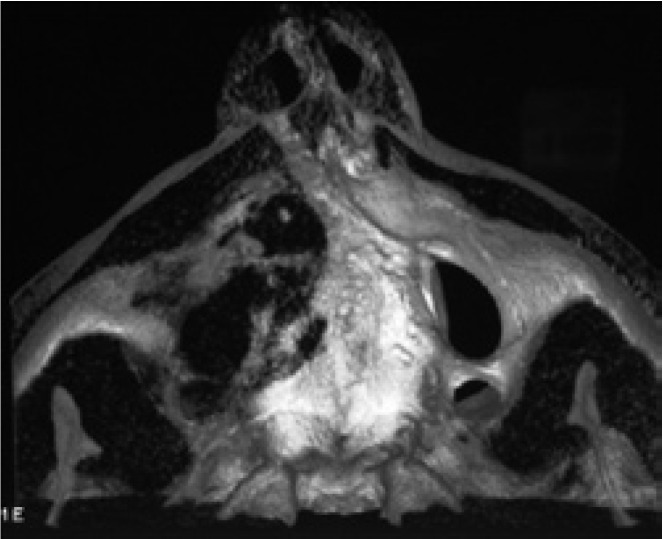
CBCT showing the borders of the lesion and the perforation areas.

A 56 year-old male patient was referred with a complaint of painless swelling in the front portion of the upper jaw. The panoramic radiographs disclosed a radiolucent mass in the right anterior region of the maxilla (Fig. 1). Due to close relation to the nasal cavity, a CBCT was required. The CBCT revealed the lesion 2.1x1.3x1.4 cm in diameter, expanding into the nasal cavity (Fig. 2). A fine-needle aspiration was performed and suggested to a cystic lesion. Under local anesthesia, the patient underwent an enucleation of the cyst, which histopathologic evaluations revealed a KCOT.

-Case 2

**Figure 3:**
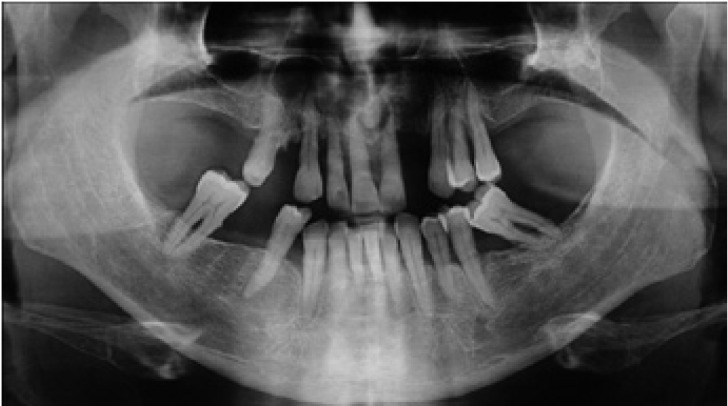
Panoramic radiograph of a KCOT occupying the right maxilla, between the right second incisor and the first premolar teeth. The border of the lesion is not readily apparent.

**Figure 4:**
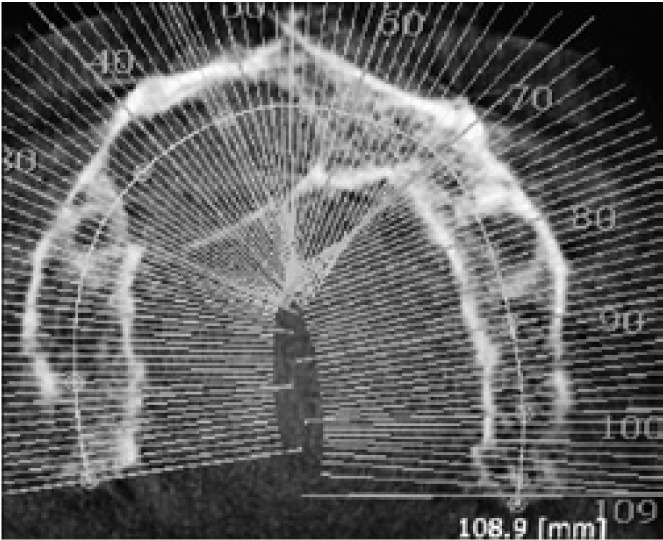
CBCT showing the borders of a KCOT. Note that the lesion shows cortical expansion at the both of the vestibular and palatal side of the maxilla.

A 38 year-old male patient complained of six months persisting pain of the right upper lateral incisor, canine and first premolar. A fine-needle aspiration revealed a cystic content. Panoramic radiographs revealed unilo-cular undefined radiolucency in the right maxillary antrum (Fig. 3). CBCT, however, showed a multilocular cystic lesion involving maxillary sinus and roots of the teeth. Lesion size was 4.5 x 3.5 x 4.5 cm (Fig. 4). Under local anesthesia the cyst was enucleated. Histopathological examinations re-vealed a KCOT.

-Case 3

**Figure 5:**
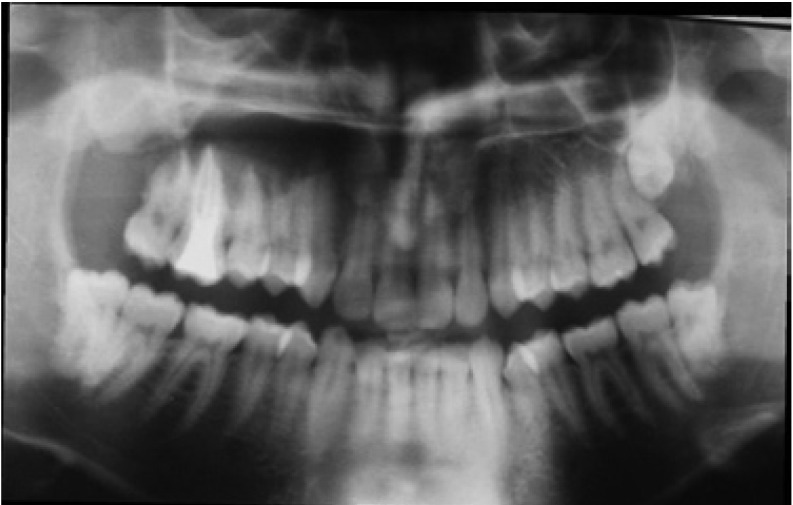
Panoramic radiograph of a KCOT occupying the right maxillary sinus, between the right second incisor and the second molar teeth. The border of the lesion is not readily apparent especially in the upper part.

**Figure 6:**
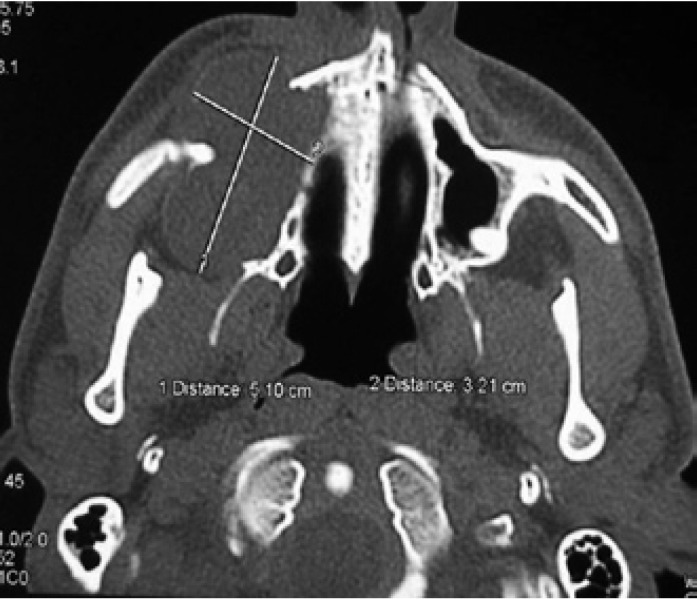
CBCT shows a large KCOT in the right maxillary sinus causing expansion of the medial and lateral sinus wall. The border of the lesion is apparent and well-circumscribed.

A 24 year-old male patient referred with complaints of pain and swelling in right maxilla. From the patient’s health history it was found out that a cyst operation in the mentioned part of maxilla was performed, a 5 years ago. Clinical examinations revealed a swelling and asymmetry below a lower eye lid from the right side of the face. From the vestibular side the soft expansion in the region of upper right premolars was noted. Panoramic radiographs revealed a radiolucent lesion extending from upper right canine to second premolar involving a maxillary antrum (Fig. 5). The lesion dimensions 3.21 x 4.3 x 5.10 cm and borders, comprising maxillary sinus below the lower edge of orbita, were analyzed by CBCT. (Fig. 6). A fine-needle aspiration find a cystic content. Preoperative diagnosis was KCOT. Under general anesthesia the apicotomy of upper right canine, premolars and first molar teeth with cyst enucleation were performed. The evaluation of submitted pathological samples revealed KCOT.

-Case 4

**Figure 7:**
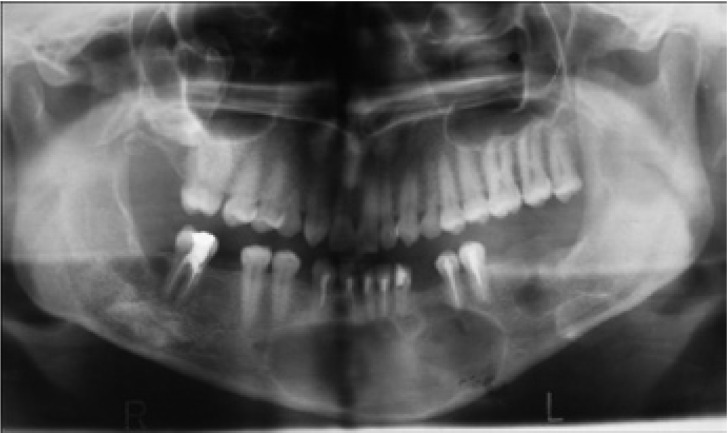
Panoramic radiograph of a KCOT occupying mandibular symphsis, between the right and left second premolars. Note the root resorptions of the anterior mandibular incisors. Slight appearance of the radiolucent areas in the right and left ramus, and in the posterior part of the right mandible associating with the second molar.

**Figure 8:**
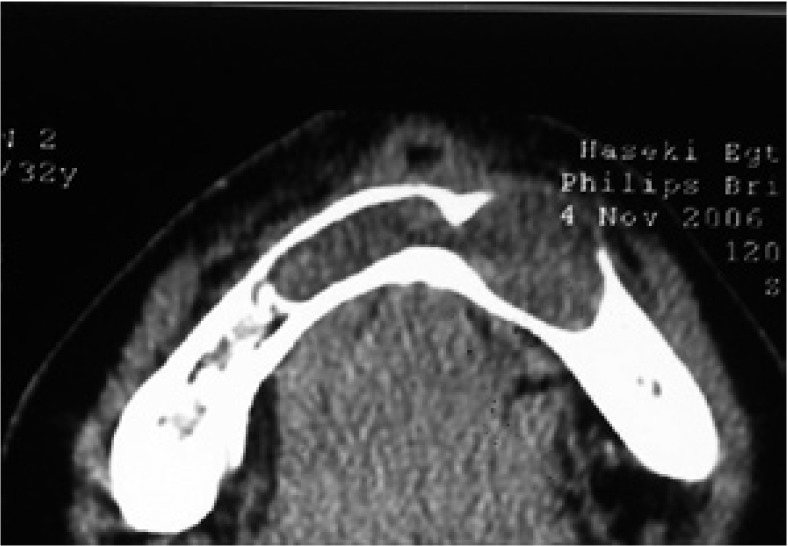
CBCT shows a large KCOT with soft tissue algorithm in the right mandibular symphysis and cortical expansion. Note scalloped border.

A 26 year-old female was referred with symptoms of swelling in anterior mandible. Panoramic radiographs showed a multiple well-circumscribed radiolucent formations: in front mandible involving a mandibular incisors and left first premolar, which undergone a root resorptions; bilaterally at mandibular body ramus area; in right maxillary sinus space, associated with impacted third molar (Fig. 7). CBCT examinations revealed a 7,4 x 3,6 x 1,8 cm in diameter cystic lesion in symphysis area, and the cystic lesions defined in previously analyzed panoramic radiographs (Fig. 8).

Due to multiple cystic formations, the patient underwent an investigations for Gorlin-Goltz syndrome, which was denied. The patient underwent enucleation of cystic lesions with extractions of impacted tooth under general anesthesia. Histopathological evaluation of the submitted samples in all cases, except maxillary lesion, revealed KCOT. The maxillary lesion was diagnosed as a dentigerous cyst.

## Discussion

Clinical occurrence of KCOTs mostly seen in mandible (65–83%) comparing with maxilla (4,5). In our described cases only in one of them, the occurence of KCOT in the mandible was reported (case 4). In the other three cases the KCOTs were located in the maxilla. These patients were also male, which once more support an opinion that the male patients show more predilection for KCOTs comparing with female patients.

Basal layer of the tumour epithelium might be budding into a supporting connective tissue, forming so called daughter cysts, which are mostly responsible for tumour reccurrences, that ranges from 5-62% and is more higher for those tumours associated with Gorlin-Goltz syndrome (5,6). Although the presented Case 4, due to multiple lesions suggested to nevoid basal cell carcinoma syndrome, after full clinical and genetic examinations, this disease was denied.

Radiographically, the KCOT is characterized as well borded radiolucent lesion with smooth and usually sclerotic margins. One of the important characteristics of KCOTs is their association with impacted teeth, with occurrence rate from 25 to 40%, which should not be neglected, due to differential diagnosis to dentigerous cysts, which was present in our described Case 4 (5,6). Other differential diagnosis may include ameloblastoma, radicular cyst, simple bone cyst, central giant cell granuloma, arteriovenous malformation, and a number of fibro-osseous lesions (12). Preoperative differentiation between these lesions is important because of the choice of surgical method. For example, ameloblastomas usually require resection and panoramic radiographs are not adequate in the differential radiologic diagnosis of these lesions (13).

Disadvantages of the panoramic radiography include geometric distortion, lack of fine detail, and numerous artifacts that require care to interpret (14). Because of these disadvantages, three dimensional investigation of the pathologic lesions in maxillofacial region became increasingly popular in oral and maxillofacial surgery (9-11). Considering the fact that conventional CT protocols are generally associated with relatively high radiation dose levels, alternative CT protocols for bone visualization and modeling are being investigated that would allow lowering the effective radiation dose for the patient, without significant loss of image quality. In this respect, CBCT holds promising potential for oral and craniofacial imaging applications. Overall advantages of the CBCT technique are a lower radiation dose, a shorter acquisition time, and reduced costs (15). Cone beam technology utilizes X-rays much more efficiently, requires far less electrical energy, and allows for the use of smaller and less expensive X-ray components than fan-beam technology. In addition, the fan-beam technology used in conventional CT scanners does not lend itself to miniaturization because it requires significant space to spiral around the entire body (16). Chau et al. (17), investigated typical absorbed doses (via a phantom) during implant imaging with conventional spiral tomography, spiral multislice CT, and CBCT. This study has quantified the absorbed dose in 5 critical organs in the oral maxillofacial region for implant imaging using 3 unique imaging modalities. For implant assessment cases using the specific modalities and systems used during this study, CBCT delivers the lowest radiation dose to the organs, whereas spiral multislice CT deli-vers the highest dose (17). The value of CBCT imaging in implant planning, surgical assessment of pathology, Temporomandibular joint (TMJ) assessment and pre- and postoperative assessment of craniofacial fractures has been reported (18). The main advantages of CBCT imaging are its accessibility, easy handling and that its offers a real-size dataset with multiplanar cross-sectional and three dimensional (3D) reconstructions based on a single scan with a low radiation (10). CBCT is also useful for diagnosis of dental abnormalities, tumours or infection with hard tissue involvement (9). Sagittal, coronal and axial CBCT images eliminate the superimposition of anatomical structures. CBCT provides additional information about the contents of the lesion (19). However, CBCT images are not so good for soft tissue display or tissues with similar densities (20).

In the present cases, it was observed that the panoramic radiograph gives more detailed information in the mandible comparing the maxilla. Especially in maxillary sinus, the panoramic radiograph was very week to describe the lesion. So we concluded that the CBCT evaluation loom large in clinical practice especially for the KCOT located in the maxillary sinus, in order to maintain a good preoperative planning.

The KCOT as a thin lining of parakeratinized epithelium, and can have thick cheesy contents due to des-quamated keratinizing squamous cells. These contents increase the radiographic attenuation of the lesion at computerized tomography (20). These features cannot be readily visualized by means of conventional 2-dimensional imaging alone (13).

The present report compared diagnostic performance of CBCT versus panoramic radiograph. Within the limits of this study, it was concluded that CBCT is more useful for the diagnosis of odontogenic keratocysts and also for proper surgical planning of the operation. CBCT provided more detailed information for evaluation of the borders of the lesion and the relation of the lesions with adjacent anatomic structures. Since KCOT has a great potential to recur, a CBCT examination is recommended for presurgical evaluation. Another important point of the present study is to emphasize that maxillofacial radiologists should be aware of the characteristic CBCT findings of the KCOT in order to differentiate the lesion from the other odontogenic lesions.
